# Feasibility and clinical implications of 3-day bladder diary derived classification of female storage lower urinary tract symptoms

**DOI:** 10.1038/s41598-022-24539-1

**Published:** 2022-11-25

**Authors:** Sheng-Mou Hsiao, Ho-Hsiung Lin

**Affiliations:** 1 Graduate School of Biotechnology and Bioengineering, Yuan Ze University, Taoyuan, Taiwan; 2 Department of Obstetrics and Gynecology, Far Eastern Memorial Hospital, Banqiao, New Taipei Taiwan; 3 Department of Obstetrics and Gynecology, National Taiwan University College of Medicine and Hospital, No. 8 Chung-Shan South Road, Taipei, 100 Taiwan

**Keywords:** Bladder, Urological manifestations

## Abstract

Our aim was to assess the feasibility of a bladder diary (BD) classification as a surrogate for urodynamic studies in women with storage lower urinary tract symptoms. A total of 3823 women who underwent urodynamic studies were reviewed. Nearly the scores of Patient Perception of Bladder Condition, Indevus Urgency Severity Scale and Overactive Bladder Symptom Score decreased gradually from the overactive bladder (OAB) wet-BD, OAB dry-BD, hypersensitive bladder (HSB) -BD, nocturia-BD to normal-BD groups (all p < 0.001). In addition, there is a trend that the rates of bladder oversensitivity decreased gradually from the OAB wet-BD, OAB dry-BD, HSB-BD, nocturia-BD to normal-BD groups (chi-square test, p < 0.001). Moreover, almost the volumes of first desire to void, normal desire to void, strong desire to void, and urgency increased gradually from the OAB wet-BD, OAB dry-BD, HSB-BD, nocturia-BD to normal-BD groups (all p < 0.001). Thus, this BD classification is correlated significantly with symptom severity, the rate of bladder oversensitivity, and bladder capacity. Nonetheless, a combination of urodynamics, clinical history, and BD is still needed for a thorough diagnosis, but that BD provides an efficient diagnosis in a proportion of patients.

## Introduction

The diagnosis of lower urinary tract dysfunctions are based on clinical symptoms, such as stress urinary incontinence (SUI) and overactive bladder syndrome (OAB). For example, SUI is defined as the complaint of involuntary loss of urine on effort or physical exertion, or on sneezing or coughing^1^. OAB is defined as the presence of urinary urgency, and usually accompanied by frequency and nocturia, in the absence of urinary tract infection or other obvious pathology^1^. However, clinical symptoms are not reliable in the differential diagnosis of lower urinary tract dysfunction in women^2^, and both history and urodynamic studies are essential for assessing complicated urinary incontinence^3^. Nonetheless, urodynamic study is an invasive procedure; thus, there is a need for a surrogate of urodynamic studies.

Bladder diary (BD) includes recording the time of each micturition, voided volume, fluid intake, episodes of urgency and incontinence, and even pad usage^1^. Two or three days of recording generally provides useful clinical data^1^. Besides, BD can be used to estimate bladder capacity, such as strong desire to void^4^, and BD has a moderate agreement with self-reported micturitions and incontinence episodes^5^. Furthermore, BD has been used as an adjuvant tool for classification [6.7]. Blaivs et al. reported the feasibility of using BD, questionnaire, uroflowmetry, and post-void residual to classify OAB into 18 subtypes^6^. Based on BD, patients with detrusor overactivity have higher voiding frequency, lower voided volume, and fewer activity-related leaks, compared with urodynamic stress incontinence^7^.

Currently, we can make a diagnosis based on the symptoms, such as OAB wet, OAB dry, and SUI. Moreover, we can make a classification from urodynamic studies. For example, Flisser et al. reported that patients can be classified into types based on the videourodynamic findings of detrusor and sphincter contractions^8^. However, to our knowledge, there is no literature mentioned about the classification of storage lower urinary tract symptoms based on BD. Thus, we aimed to elucidate the feasibility of a BD derived classification by analyzing the correlations between this BD classification and the clinical/urodynamic diagnosis and parameters.

## Results

**Figure 1:**
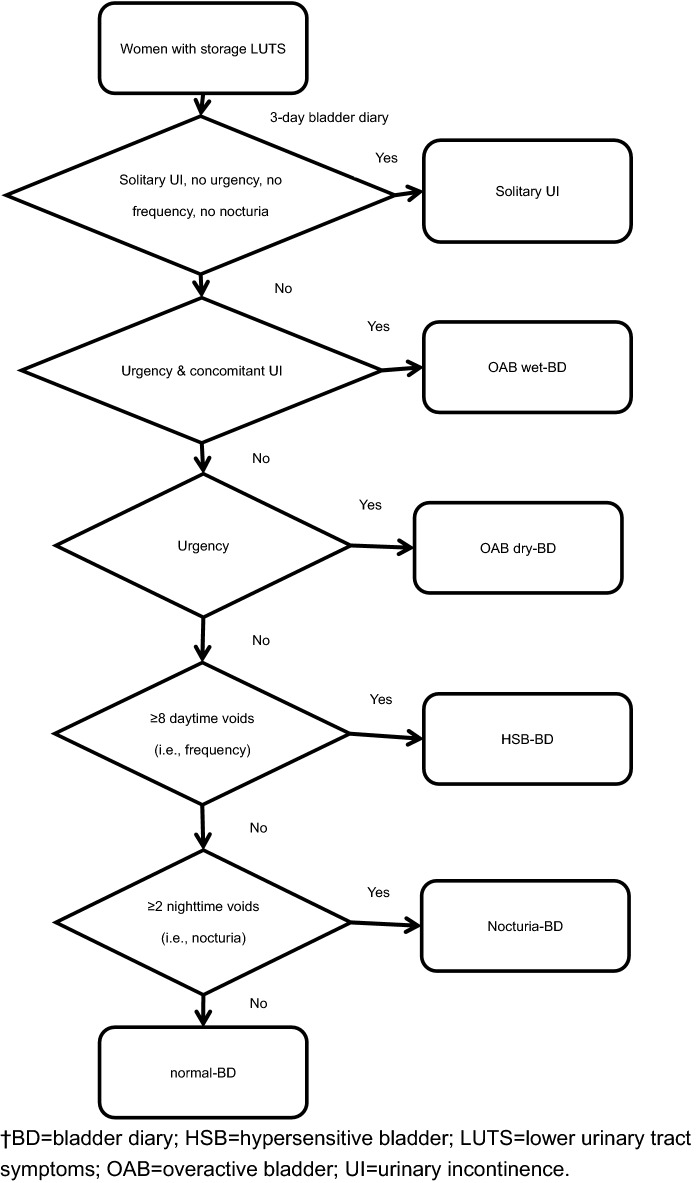
Flow chart of classifying groups according to the 3-day bladder diary.

After excluding women with solitary urinary incontinence (n = 128), a total of 3823 women were reviewed (Fig. 1, Table 1). In women with OAB (i.e., diagnosed by the complaint of urinary urgency^1^, n = 2127), the percentages of the OAB wet-BD, OAB dry-BD, hypersensitive bladder (HSB) -BD, nocturia-BD and normal-BD groups were 20.5% (436/2127), 53.8% (1145/2127), 15.0% (320/2127), 8.2% (179/2127) and 2.2% (47/2127), respectively (Table 1).

**Table 1:** Comparison of clinical diagnoses and severity of clinical symptoms among the five groups (n = 3823).

Variables	All cases	OAB wet-BD	OAB dry-BD	HSB-BD	Nocturia-BD	Normal-BD	p^†^	Post hoc analysis^‡^			
	(n = 3823)	(n = 637, a)	(n = 1903, b)	(n = 690, c)	(n = 418, d)	(n = 175, e)		a	b	c	d
								Compared with:			
Age (years)	60.7 ± 12.4	62.6 ± 12.6	59.4 ± 12.6	61.8 ± 11.6	62.9 ± 11.8	58.0 ± 12.0	< 0.0001	be	cd	e	e
SUI	2429 (64)	509 (80)	1175 (62)	392 (57)	241 (58)	112 (64)	< 0.001	bcde	c	–	–
OAB	2127 (56)	436 (69)	1145 (60)	320 (46)	179 (43)	47 (27)	< 0.001	bcde	cde	e	e
VD	234 (6)	28 (4)	123 (6)	61 (9)	19 (5)	3 (2)	< 0.001	c	ce	de	–
POP	1703 (45)	182 (29)	739 (39)	387 (56)	283 (68)	112 (64)	< 0.001	bcde	cde	d	–
PPBC	3.2 ± 1.5	4.1 ± 1.3	3.4 ± 1.4	2.9 ± 1.4	2.2 ± 1.3	1.9 ± 1.0	< 0.0001	bcde	cde	de	–
USS	1.7 ± 1.1	2.5 ± 1.2	1.8 ± 1.0	1.3 ± 0.9	1.0 ± 0.9	0.8 ± 0.9	< 0.0001	bcde	cde	de	–
OABSS	6.0 ± 3.6	9.5 ± 3.0	6.5 ± 3.1	4.2 ± 2.7	3.1 ± 2.3	1.8 ± 2.1	< 0.0001	bcde	cde	de	e

Values are expressed by mean ± standard deviation or number (percentage), and the numerator was the patient number of the coexistent variable, and the denominator was the patient number of the whole population or subgroup.

*BD* bladder diary, *HSB* hypersensitive bladder, *OAB* overactive bladder syndrome, *OABSS* overactive bladder symptom score, *POP* pelvic organ prolapse, *PPBC* Patient Perception of Bladder Condition, *SUI* stress urinary incontinence, *USS* the Indevus Urgency Severity Scale, *VD* voiding dysfunction.

^†^Analysis of Variances.

^‡^a = OAB wet-BD, b = OAB dry-BD, c = HSB-BD, d = nocturia-BD, e = normal-BD. Post hoc analyses were performed with Bonferroni correction. Only those groups with significant between-group differences (i.e., p < 0.05) were shown here, and their group names were filled in the cell of the corresponding row.

In women with pelvic organ prolapse (i.e., diagnosed by pelvic examination, n = 1703), the percentages of the OAB wet-BD, OAB dry-BD, HSB-BD, nocturia-BD and normal-BD groups were 10.7% (182/1703), 43.4% (739/1703), 22.7% (387/1703), 16.6% (283/1703) and 6.6% (112/1703), respectively (Table 1).

**Figure 2:**
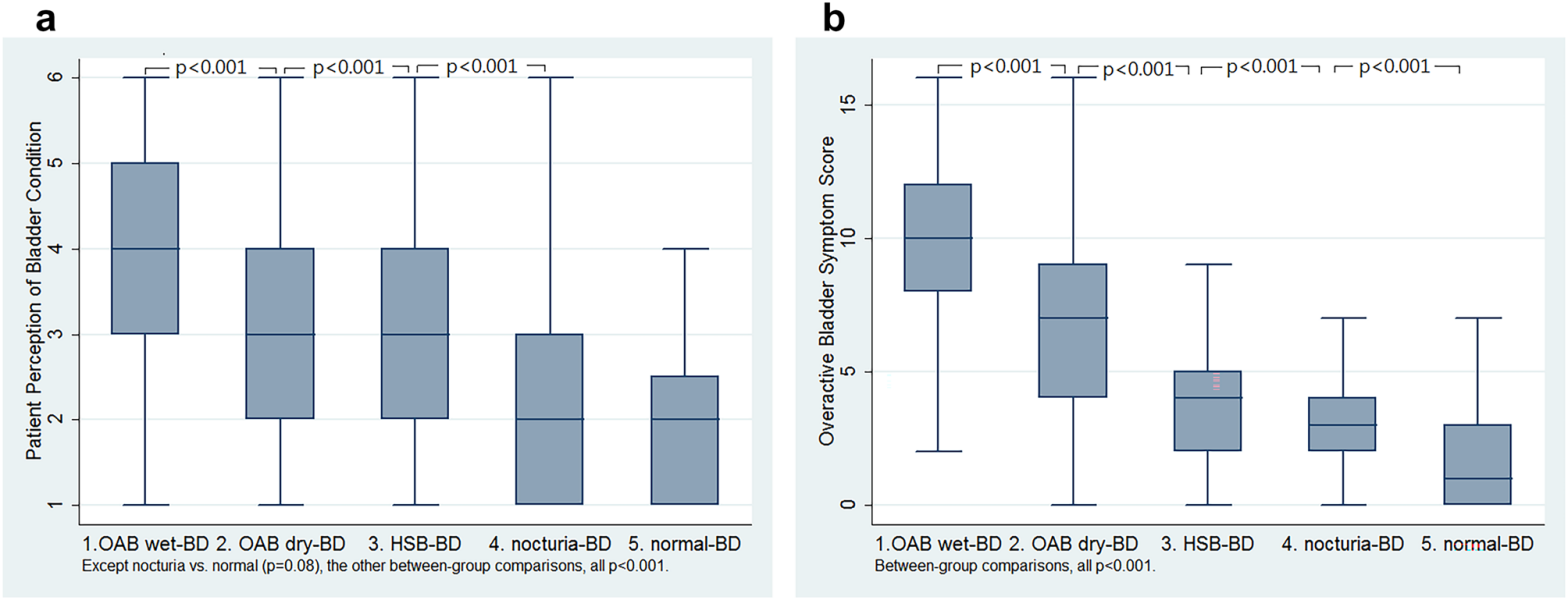
Box plots of (**a**) the score of Patient Perception of Bladder Condition (PPBC) and (**b**) the score of Overactive Bladder Symptom Score (OABSS) between the five groups (only those comparisons with p < 0.05 between the adjacent groups were shown in the figures).

Symptom severity of OAB (i.e., the scores of Patient Perception of Bladder Condition, the Indevus Urgency Severity Scale and Overactive Bladder Symptom Score (OABSS)) decreased gradually from the OAB wet-BD, OAB dry-BD, HSB-BD, nocturia-BD to normal-BD groups (all p < 0.0001, Fig. 2a,b, Table 1).

**Figure 3:**
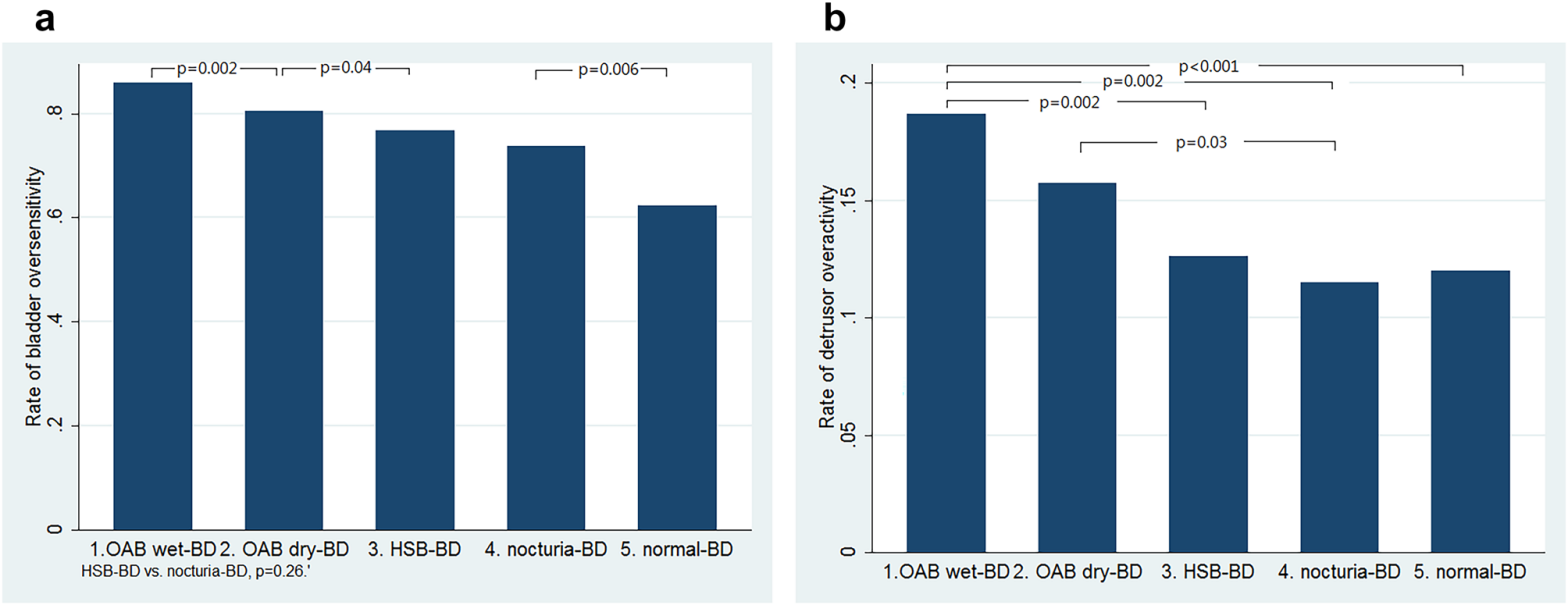
(**a**) Comparison of percentages of bladder oversensitivity between the five groups (only those comparisons with p < 0.05 between the adjacent groups were shown in the figure), and (**b**) comparison of percentages of detrusor overactivity between the five groups.

The rates of bladder oversensitivity decreased gradually from the OAB wet-BD, OAB dry-BD, HSB-BD, nocturia-BD to normal-BD groups, despite there was no difference between the HSB-BD and nocturia-BD groups (p = 0.26, Fig. 3a, Table 2).

**Table 2:** Comparison of urodynamic diagnoses among the five groups (n = 3823).

Variables	All cases	OAB wet-BD	OAB dry-BD	HSB-BD	Nocturia-BD	Normal-BD	p^†^	Post hoc analysis^‡^			
	(n = 3823)	(n = 637, a)	(n = 1903, b)	(n = 690, c)	(n = 418, d)	(n = 175, e)		a	b	c	d
								Compared with:			
BO	3117 (82)	547 (86)	1530 (80)	529 (77)	308 (74)	109 (62)	< 0.001	bcde	cde	e	e
DO	504 (13)	119 (19)	299 (16)	87 (13)	48 (11)	21 (12)	0.003	cde	d	–	–
USI	2246 (59)	497 (78)	1112 (58)	358 (52)	191 (46)	88 (50)	< 0.001	bcde	cde	d	–
BOO	92 (2)	14 (2)	50 (3)	14 (2)	10 (2)	4 (2)	0.92	–	–	–	–

Values are expressed by mean ± standard deviation or number (percentage), and the numerator was the patient number of the coexistent variable, and the denominator was the patient number of the whole population or subgroup.

*BD* bladder diary, *BO* bladder oversensitivity, *BOO* bladder outlet obstruction, *DO* detrusor overactivity, *HSB* hypersensitive bladder, *OAB* overactive bladder syndrome, *USI* urodynamic stress incontinence.

^†^Analysis of Variances.

^‡^a = OAB wet-BD, b = OAB dry-BD, c = HSB-BD, d = nocturia-BD, e = normal-BD. Post hoc analyses were performed with Bonferroni correction. Only those groups with significant between-group differences (i.e., p < 0.05) were shown here, and their group names were filled in the cell of the corresponding row. For example, if there was a statistical difference between the a and b groups, then “b” was filled in the cell of the “a compared with” row.

The rate of detrusor overactivity in the OAB wet-BD group was higher, compared with HSB-BD, nocturia-BD and normal-BD (Fig. 3b), and the rate of detrusor overactivity in the OAB dry-BD group was higher than the nocturia-BD group (Fig. 3b, Table 2).

**Figure 4:**
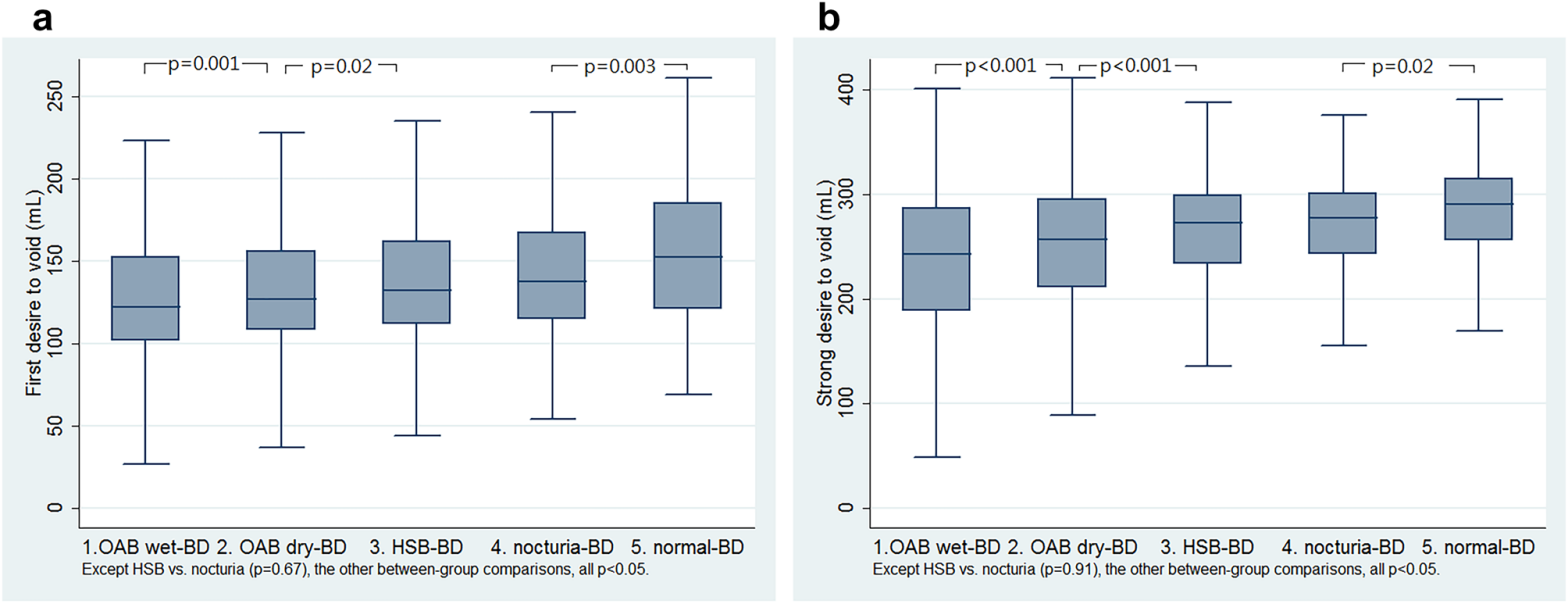
Box plots of (**a**) first desire to void (mL) and (**b**) strong desire to void (mL) between the five groups (only those comparisons with p < 0.05 between the adjacent groups were shown in the figures).

The volumes of first desire to void, normal desire to void, strong desire to void, and urgency increased gradually from the OAB wet-BD, OAB dry-BD, HSB-BD, nocturia-BD to normal-BD groups (all p < 0.0001, Fig. 4a,b, Table 3).

**Table 3:** Comparison of urodynamic parameters among the five groups (n = 3823).

Variables	All cases	OAB wet-BD	OAB dry-BD	HSB-BD	Nocturia-BD	Normal-BD	p^†^	Post hoc analysis^‡^			
	(n = 3823)	(n = 637, a)	(n = 1903, b)	(n = 690, c)	(n = 418, d)	(n = 175, e)		a	b	c	d
								Compared with:			
VV (mL)	266 ± 127	248 ± 167	265 ± 126	268 ± 123	279 ± 128	308 ± 145	< 0.0001	bde	e	e	–
PVR (mL)	42 ± 38	40 ± 34	43 ± 40	46 ± 42	37 ± 28	37 ± 31	0.001	–	d	d	–
FD (mL)	136 ± 37	127 ± 38	133 ± 36	138 ± 36	143 ± 36	154 ± 39	< 0.0001	bcde	cde	e	e
ND (mL)	197 ± 45	184 ± 48	195 ± 44	202 ± 39	210 ± 41	225 ± 46	< 0.0001	bcde	cde	de	e
SD (mL)	254 ± 57	234 ± 62	251 ± 57	263 ± 50	269 ± 50	285 ± 50	< 0.0001	bcde	cde	e	e
Urgency (mL)	333 ± 80	308 ± 86	330 ± 80	345 ± 74	350 ± 73	373 ± 71	< 0.0001	bcde	cde	e	e
PdetQmax (cmH_2_O)	26.6 ± 16.7	26.2 ± 17.1	26.7 ± 16.3	25.4 ± 15.1	27.2 ± 20.0	29.7 ± 15.8	0.06	–	–	–	–
MUCP (cmH_2_O)	64.0 ± 32.6	57.6 ± 31.8	66.6 ± 33.2	66.6 ± 32.0	59.4 ± 30.2	60.5 ± 31.7	< 0.0001	bc	d	d	–

Values are expressed by mean ± standard deviation.

*BD* bladder diary, *FD* first desire to void, *HSB* hypersensitive bladder, *MUCP* maximum urethral closure pressure, *ND* normal desire to void, *OAB* overactive bladder syndrome, *PdetQmax* detrusor pressure at maximum flow rate, *PVR* post-void residual, *Qmax* maximum flow rate, *SD* strong desire to void, *VV* voided volume.

^†^Analysis of Variances.

^‡^a = OAB wet-BD, b = OAB dry-BD, c = HSB-BD, d = nocturia-BD, e = normal-BD. Post hoc analyses were performed with Bonferroni correction. Only those groups with significant between-group differences (i.e., p < 0.05) were shown here, and their group names were filled in the cell of the correspondin grow.

**Figure 5:**
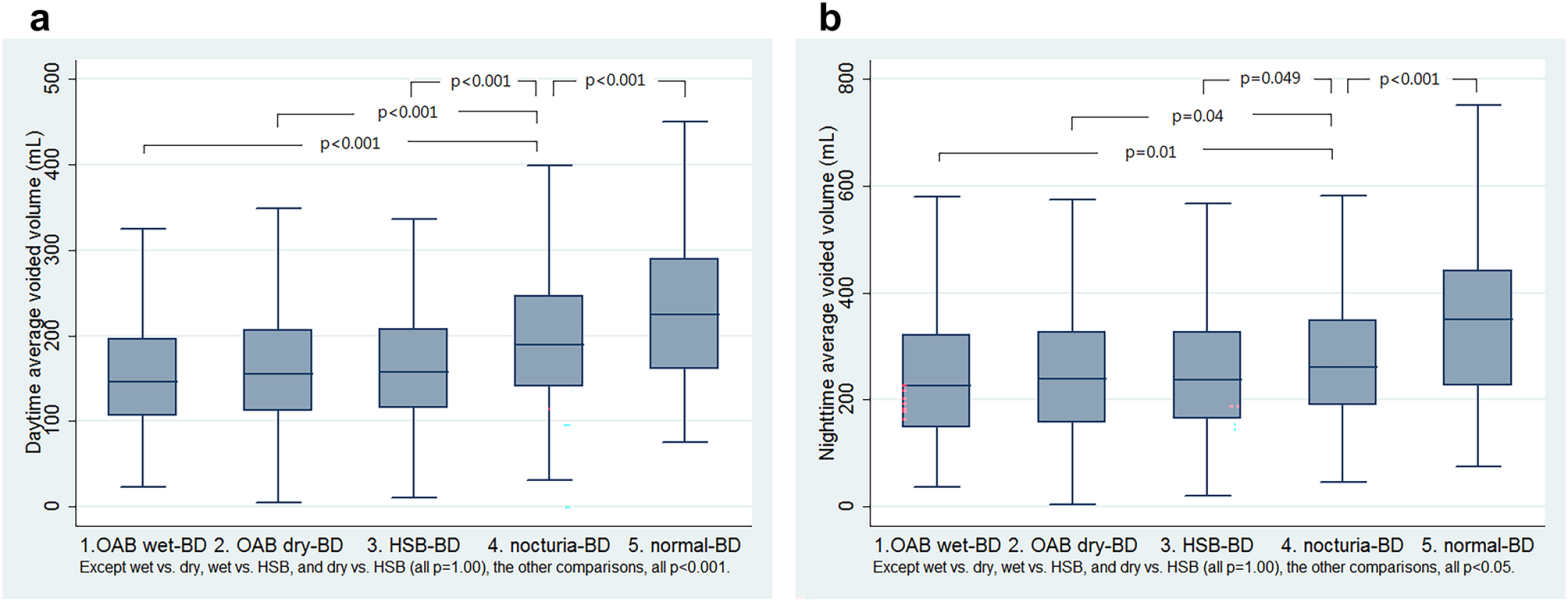
Box plots of (**a**) daytime average voided volume (mL) and (**b**) nighttime average voided volume (mL) between the five groups.

The BD-nocturia group had larger daytime and nighttime average voided volumes, compared with OAB wet-BD, OAB dry-BD and HSB-BD (Fig. 5a,b, Table 4). The nocturia-BD group had a higher percentage of nocturnal polyuria, compared with the other four groups (Table 4).

**Table 4:** Comparison of bladder diary parameters among the five groups (n = 3823).

Variables	All cases	OAB wet-BD	OAB dry-BD	HSB-BD	Nocturia-BD	Normal-BD	p^†^	Post hoc analysis^‡^			
	(n = 3823)	(n = 637, a)	(n = 1903, b)	(n = 690, c)	(n = 418, d)	(n = 175, e)		a	b	c	d
								Compared with			
DTFI (mL, 24 h)	1529 ± 590	1512 ± 647	1548 ± 628	1611 ± 604	1350 ± 509	1490 ± 526	< 0.0001	cd	d	d	–
NTFI (mL, 24 h)	202 ± 179	220 ± 197	202 ± 177	203 ± 173	207 ± 178	112 ± 119	< 0.0001	e	e	e	e
TFI (mL, 24 h)	1731 ± 665	1732 ± 693	1750 ± 684	1814 ± 655	1557 ± 547	1602 ± 559	< 0.0001	d	de	de	–
DAVV (mL)	172 ± 77	161 ± 77	166 ± 75	165 ± 66	199 ± 80	234 ± 96	< 0.0001	de	de	de	e
NAVV (mL)	262 ± 132	250 ± 135	257 ± 132	254 ± 123	277 ± 115	351 ± 157	< 0.0001	de	de	de	e
AVV (mL)	190 ± 81	179 ± 81	184 ± 80	181 ± 69	221 ± 82	252 ± 98	< 0.0001	de	de	de	e
DTVV (mL, 24 h)	1299 ± 590	1289 ± 610	1314 ± 602	1440 ± 572	1049 ± 458	1202 ± 546	< 0.0001	cd	cd	de	e
NTVV (mL, 24 h)	536 ± 280	553 ± 292	542 ± 278	525 ± 270	590 ± 281	321 ± 179	< 0.0001	e	de	de	e
TVV (mL, 24 h)	1835 ± 724	1842 ± 766	1857 ± 730	1965 ± 696	1635 ± 635	1524 ± 629	< 0.0001	cde	cde	de	–
NP (n = 3823)	2477 (65)	402 (63)	1284 (67)	367 (53)	350 (84)	74 (42)	< 0.001	bcde	cde	de	e
NP in OAB (n = 2127)	1410 (66)	273 (63)	777 (68)	185 (58)	154 (86)	21 (45)	< 0.001	bcde	cde	de	e

Values are expressed by mean ± standard deviation or number (percentage), and the numerator was the patient number of the coexistent variable, and the denominator was the patient number of the whole population or subgroup.

*AVV* average voided volume, *BD* bladder diary, *DAVV* daytime average voided volume, *DTFI* volume of daytime total fluid intake, *DTVV* daytime total voided volume, *HSB* hypersensitive bladder, *NAVV* nighttime average voided volume, *NP* nocturnal polyuria, *NTFI* volume of nighttime total fluid intake, *NTVV* nighttime total voided volume, *OAB* overactive bladder syndrome, *TFI* volume of total fluid intake, *TVV* total voided volume.

^†^Analysis of Variances or chi-squared test.

^‡^a = OAB wet-BD, b = OAB dry-BD, c = HSB-BD, d = nocturia-BD, e = normal-BD. Post hoc analyses were performed with Bonferroni correction or chi-squared test. Only those groups with significant between-group differences (i.e., p < 0.05) were shown here, and their group names were filled in the cell of the corresponding row. For example, if there was a statistical difference between the a and b groups, then “b” was filled in the cell of the “a compared with” row.

The nocturia-BD group had a lower volume of daytime total fluid intake, compared with OAB wet-BD, OAB dry-BD and HSB-BD (Table 4). The volume of nighttime fluid intake did not differ between the OAB wet-BD, OAB dry-BD, HSB-BD and nocturia-BD groups (Table 4).

## Discussion

Based on symptoms, women with storage lower urinary tract symptoms have been classified into OAB wet, OAB dry, and HSB to represent a spectrum of disease severity^9,10^. In this study, the symptom severity decreased gradually from the OAB wet-BD, OAB dry-BD, HSB-BD, nocturia-BD to normal-BD groups (Fig. 2a,b, Table 1). In addition, the rates of bladder oversensitivity decreased gradually from the OAB wet-BD, OAB dry-BD to HSB-BD groups (Fig. 3a, Table 2). Moreover, the bladder capacity increased gradually from the OAB wet-BD, OAB dry-BD, HSB-BD, nocturia-BD to normal-BD groups (Fig. 4a,b, Table 3). Thus, based on BD, classifying storage lower urinary tract symptoms seems to be feasible and reasonable to represent a spectrum of disease severity.

Based on urodynamic studies, OAB women can be diagnosed to have bladder oversensitivity and/or detrusor overactivity (Table 2). The Spearman’s rho between bladder oversensitivity and OABSS is 0.21 (p < 0.0001), and the Spearman’s rho between detrusor overactivity and OABSS is 0.05 (p = 0.001). In addition, the Spearman’s rho between strong desire to void (mL) and OABSS is − 0.35 (p < 0.0001), and the Spearman’s rho between urgency (mL) and OABSS is − 0.33 (p < 0.0001). That is, the correlations between urodynamic parameters/diagnosis and symptom severity are weak.

Contrarily, according to the fact of a decrease of OABSS from OAB wet-BD to normal-BD (Table 1), we may arbitrarily define the ranks of symptom severity of OAB wet-BD, OAB dry-BD, HSB-BD, nocturia-BD and normal-BD are 5, 4, 3, 2, and 1, respectively; and then the Spearman’s rho between the BD classification and OABSS is 0.60 (p < 0.0001). That is, the correlation between the BD classification and symptom severity is strong. Therefore, this BD classification seems to have a better correlation with symptom severity, compared with urodynamic diagnosis. In addition, it has been reported that there is no correlation between normalized urodynamic findings and the changes of OABSS after OAB treatment^11^. Thus, the role of urodynamic studies in the treatment of female OAB seems limited. Instead, we cannot overlook the importance of BD in the era of precision medicine.

Symptom based diagnoses are based on recall memories about a history of recent weeks or months; and this may not represent the real symptom severity of a recent urinary condition and lead to under- or undue treatment. This BD classification could exhibit subjective and objective urinary severity in the recent three days and may alter presumed treatment. For instance, the main medical treatments for OAB include antimuscarinics, beta 3-agonist, or combination therapy. Nonetheless, a total of 8.2% (179/2127) of OAB women were allocated into the nocturia-BD group (Table 1), and women in the nocturia-BD group may be better treated by desmopressin^12,13^, instead of antimuscarinics or beta-3 agonist. In addition, prolonged treatment was suggested to decrease retreatment in OAB women with small bladder capacity^14^. The OAB wet-BD group has the smallest bladder capacity (Fig. 4a,b, Table 3). Thus, women in the OAB wet-BD group may be treated with prolonged treatment to decrease retreatment^11^.

Combination treatment (i.e., beta-3 agonist plus antimuscarinics) has been found to have an additive effect, compared with monotherapy^15,16^. In the era of precision medicine, classifying individuals into subpopulations has been suggested for OAB patients^17^. Therefore, treatment can be focused on those who will benefit, and sparing expenses and side effects can be expected for those who will not^17^. Thus, we may treat our OAB patients according to this BD classification. For example, we may treat our OAB wet-BD women with combined therapy, OAB dry-BD women with high-dose monotherapy (e.g., 50 mg mirabegron), HSB-BD women with low-dose monotherapy (e.g., 25 mg mirabegron), nocturia-BD women with desmopressin, and normal-BD with lifestyle modifications as their frontline treatment. Future researches are suggested to be carried out to justify this BD classification.

In this study, we did find a higher rate of nocturnal polyuria in the nocturia-BD group (Table 4), and this finding is in line with the concept of nocturnal polyuria as one of the main causes of nocturia^12^. Thus, this BD classification system seems to be a reasonable tool for classifying the nocturia-BD group in women with storage lower urinary tract symptoms.

Coexistent prevalence rates of urgency and OAB symptoms in prolapse women have been reported to be 49.7% and 69.4%, respectively^18^. Similarly, the percentages of the OAB wet-BD and OAB dry-BD groups were 10.7% (182/1703), and 43.4% (739/1703) in women with pelvic organ prolapse, respectively (Table 1). Furthermore, baseline coexistent OAB is considered a risk factor for postoperative OAB^19^, and women with severe prolapse have a higher risk of persistent urgency incontinence or frequency after prolapse surgery^20^. Besides, urgency and frequency are more likely to improve after prolapse repair, compared with other symptoms^21^. Thus, the awareness of the coexistent BD groups should be helpful in perioperative consultation about persistent OAB after prolapse surgery.

It is worth mentioning that there are discordances between BD and self-reported severity. Hikita et al. reported that self-reported score overestimates daytime frequency and nocturia, compared with BD derived score^22^. In addition, it has been reported that high correlations between changes in the OABSS items and the corresponding BD variables are only found for urgency incontinence and nocturia^23^. In our study, the diagnosis of OAB was made according to the complaint of urinary urgency. The OAB wet-BD and OAB dry-BD groups were defined by the presence of urinary urgency in the BD. Theoretically, the patient number of the OAB wet-BD and OAB dry-BD groups should be equal to that of OAB. Nonetheless, only 74.3% (1581/2127) women with OAB were allocated to the OAB wet-BD or OAB dry-BD groups. Missing data or less frequent occurrence of urinary urgency (i.e., urinary urgency did not occur in three days while recording the BD) might explain the above discrepancy.

Injury to autonomic nerves may occur in radical hysterectomy or advanced gynecologic oncologic surgery (e.g., pelvic exenteration and laterally extended endopelvic resection)^24–26^. Pelvic splanchnic nerve, which runs beneath the deep uterine vein, may be damaged during the removal of the cardinal ligament. Hypogastric nerve, which runs within the connective tissue of the lateral rectal wall, may be injured during the division of the uterosacral ligament^26^. Inferior hypogastric plexus may be damaged during the division of the paracolpium^26^. An injury to the pelvic splanchnic nerve may deteriorate bladder emptying, and damage to the hypogastric nerve may impair detrusor relaxation. Preoperative BD classification may reveal baseline information about bladder storage function and be helpful in postoperative consultation.

Among the compartments of pelvic organ prolapse, anterior compartment prolapse (i.e., cystocele) represents the most challenging condition. Cystocele can be treated by native tissue repair or transvaginal mesh surgery^27,28^. Transvaginal mesh surgery is considered to have a higher anatomical success rate and fewer recurrences; and native tissue repair is considered to have fewer complications^27–31^. Currently, transvaginal mesh surgery is not recommended for primary repair due to mesh complications.

It has been reported that reoperation for pelvic organ prolapse was common (about 30% of cases), and the time intervals between repeated procedures decreased with each successive repair^32^. Transvaginal mesh was associated with a lower recurrence rate of cystocele, compared with native tissue repair^31^. However, transvaginal mesh failed to decrease the recurrence rate in women with previous prolapse surgery, compared with native tissue repair^33,34^. Thus, the issue of whether using transvaginal mesh to decrease prolapse recurrence in women with previous repair remains undetermined.

Limitation of this study includes a retrospective nature. In addition, the percentages of bladder outlet obstruction did not differ between the five groups (Table 1). For women with voiding dysfunction, subsequent urodynamic or videourodynamic studies may be needed for precise diagnosis.

In conclusion, a classification based on BD to classify female storage lower urinary tract symptoms is established, and this BD classification is correlated significantly with symptom severity and bladder capacity. Nonetheless, a combination of urodynamics, clinical history, and BD is needed for a thorough diagnosis, but that BD provides an efficient diagnosis in a proportion of patients.

## Methods

Between July 2009 and December 2020, all women with lower urinary tract symptoms or pelvic organ prolapse, who underwent urodynamic studies, were included in this retrospective study. Those women who did not have complete data or only solitary urinary incontinence in the BD were excluded (Fig. 1). BD, Patient Perception of Bladder Condition^35^, the Indevus Urgency Severity Scale^36^, OABSS^37^, Urinary Distress Inventory, Incontinence Impact Questionnaire, and urodynamic studies were reviewed. Patient Perception of Bladder Condition is a global assessment of bladder condition among patients with OAB^35^. The Indevus Urgency Severity Scale is a measure of urgency severity^36^. The OABSS is the sum score of four overactive bladder symptoms (i.e., daytime frequency, nocturia, urgency and urgency incontinence)^37^. The Urogenital Distress Inventory is used to assess symptom distress. The Incontinence Impact Questionnaire is used to assess the impact of urinary incontinence on quality of life. The contents of BD include nighttime and daytime voids, voided volume per micturition, volume of fluid intake, and episodes of urgency and incontinence. Our BD is similar to that of Hikita et al.^22^. National Taiwan University Hospital Research Ethics Committee approved the study protocol (No. 202105069RINA). All methods were carried out in accordance with relevant guidelines and regulations. The National Taiwan University Hospital Research Ethics Committee approved to waive the requirement to obtain informed consent from the enrolled patients. This study conformed to the Enhancing the QUAlity and Transparency Of health Research (EQUATOR) network guidelines.

Based on the 3-day BD, those women with solitary urinary incontinence without coexistent urgency were excluded from further analysis. The enrolled women were allocated into the following five groups according to the findings of the 3-day BD (Fig. 1). (1) OAB wet-BD was defined if having at least one episode of urgency incontinence (i.e., the presence of concomitant urgency and urinary incontinence in the same period). (2) OAB dry-BD was defined if having at least one episode of urgency and without concomitant urinary incontinence. (3) HSB-BD was defined if having ≥ 8 daytime voids in at least one day of the 3-day BD. (4) Nocturia-BD was defined if having ≥ 2 nighttime voids in at least one day of the 3-day BD, which resulted in sleep interruption due to the need of micturition, and < 8 daytime voids in every day of the 3-day BD. (5) The other women were allocated into the normal-BD group (Fig. 1).

Urodynamic studies were performed with a Life-Tech six-channel monitor with computer analysis and Urolab/Urovision System V (Houston, Texas, USA), with patients in a seated position. The studies included uroflowmetry, both filling (at a rate of 60 mL/min) and voiding cystometry with infusion of 35 °C distilled water, and stress urethral pressure profiles with a full amount of distilled water in the bladder. All terminology used in this paper conforms to the standards recommended by the International Continence Society^1^. All procedures were performed by an experienced technician and the data were interpreted by a single observer (HHL) to avoid interobserver variability. Nocturnal polyuria was defined as a daytime voided volume > 33% of the daily total voided volume in women ≥ 65 years old, and > 20% of the daily total voided volume in women < 65 years old^38,39^. Bladder outlet obstruction was defined when the detrusor pressure at maximum flow was greater than or equal to 40 cmH_2_O, and the maximum flow rate was less than 12 mL/s^40,41^. A volume of strong desire to void < 350 mL was classified as having bladder oversensitivity^10,42^.

Stata software (Version 11.0; Stata Corp, College Station, Texas, USA) was used for statistical analyses. Analysis of variance, chi-square test, and Fisher’s exact test were used as appropriate. Post hoc analyses by Bonferroni test were performed only in those comparisons with p < 0.05 after analysis of variance. A p value of less than 0.05 was considered statistically significant. The main objective of this study was to assess the feasibility of this BD classification by its relationship with symptom severity and urodynamic diagnosis and parameters.

## Data Availability

The datasets generated during and/or analyzed during the current study are available from the corresponding author on reasonable request.
